# Supplementary material for: Spatio-temporal networks: reachability, centrality and robustness
Source: R Soc Open Sci. 2016 Jun 29;3(6):160196. doi: 10.1098/rsos.160196 (PMC4929911; doi:10.1098/rsos.160196)
Supplement: Electronic Supplementary Material_Williams Musolesi_2016_RSOS.pdf Dataset descriptors and visualisations. This document provides further detail on the data materials used to construct each network explored in the paper. The supplementary material includes URLs to data materials we have made availabl [file rsos160196supp1.pdf]

Spatio-temporal networks: reachability, centrality, and robustness (Supplementary material)

Matthew J. Williams[1,2,*] and Mirco Musolesi[2,1]

[1] *School of Computer Science, University of Birmingham, Edgbaston B15 2TT, UK*

[2] *Department of Geography, University College London, London WC1E 6BT, UK*

1. EMPIRICAL DATASETS

a. Data sources for network construction

In the interest of reproducibility, here we detail the publicly available data sources used to construct the four spatio-temporal networks used in this work.

London Metro: Comprehensive data on timetabled journeys by London Underground trains each week is provided by *Transport for London* (TfL) at the TfL Open Data Hub[1]. These are provided in TransXChange[2] format, an XML-based standard maintained by the UK Department for Transport.

Paris Metro: Data on timetabled Paris Metro journeys each week is provided by *Régie Autonome des Transports Parisiens* (RATP) at the RATP Open Data website[3]. These are provided in GTFS format[4].

New York Metro: Data on timetabled New York City Subway journeys each week is provided in GTFS format by the *Metropolitan Transportation Authority* (MTA) at the MTA Developer Data Downloads website[5]. The overall MTA GTFS covers both New York City Subway and the Staten Island Railway, comprising 440 stations. Reducing this to New York City Subway stations active in the chosen observation window yields a network of 417 nodes.

US Flights: Flight take-off and landing times are compiled by the US Department of Transportation[6]. Flights are provided in monthly CSV (comma-separated values) files.

C. Elegans: Coordinates of neurons in the adult worm are provided by the Dynamic Connectome Lab[7] and described in Ref. [1]. Neuronal connectivity data are available at WormAtlas[8] and were originally compiled from the work in Ref. [2] and Ref. [3].

StudentLife: The complete StudentLife dataset has been made publicly available by the Dartmouth StudentLife Team[9]. The dataset is described in Ref. [4].

b. Geospatial embedding

London Metro, US Flights, Paris Metro, New York Metro, and StudentLife represent geospatial systems. Here we describe how we obtained the geospatial embedding of these data.

For the London Metro network, we associated Underground stations in the *Transport for London* Journey Planner Timetable dataset with their corresponding geographic coordinates in the UK NaPTAN transport stop metadata[10], giving nodes embedded in the WGS-84 geodetic coordinate space. We merged multiple platforms corresponding to the same station into one single entity, yielding 270 stations overall. Each station is represented by the centroid of its one-or-more platforms. We calculate geospatial distance over the surface of the Earth using the Vincenty formula.

Station and sub-station geo-coordinates are provided in the GTFS data for Paris Metro and New York Metro. We reconcile Paris Metro sub-stations to a single station by station name. New York Metro sub-stations are unified by GTFS parent station.

For US Flights, airport geographic coordinates are provided in the Aviation Master Coordinate Table[11].

Geo-location in StudentLife is obtained by the WGS-84 geographic coordinates provided by each student's Android smartphone. The Android API tracks location via a mixture of WiFi geo-location, GPS, and and cellular

* Author for correspondence. E-mail: m.j.williams@cs.bham.ac.uk

[1] Available on 05/05/2016. `http://www.tfl.gov.uk/info-for/open-data-users/`

[2] `https://www.gov.uk/government/collections/transxchange`

[3] Available on 05/05/2016. `http://www.ratp.fr/fr/ratp/r_70350/open-data/`

[4] Available on 05/05/2016. `http://dataratp.opendatasoft.com/explore/dataset/offre-transport-de-la-ratp-format-gtfs/`

[5] Available on 05/05/2016. `http://web.mta.info/developers/download.html`

[6] Available on 05/05/2016. `http://www.rita.dot.gov/bts`

[7] Available on 05/05/2016. `http://www.dynamic-connectome.org`

[8] `http://www.wormatlas.org`

[9] Available on 05/05/2016. `http://studentlife.cs.dartmouth.edu`

[10] UK National Public Transport Access Nodes (NaPTAN) metadata: `http://data.gov.uk/dataset/naptan`. Available on 05/05/2016.

[11] The Master Coordinate Table is among the aviation support metadata managed by the US Department of Transportation: `http://www.transtats.bts.gov`. Available on 05/05/2016.

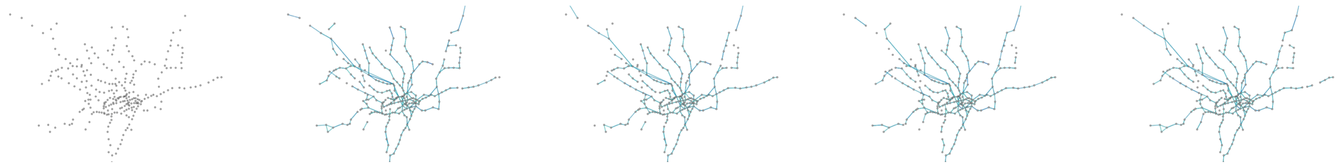

(a) London Metro (04:00 - 20:00 GMT)

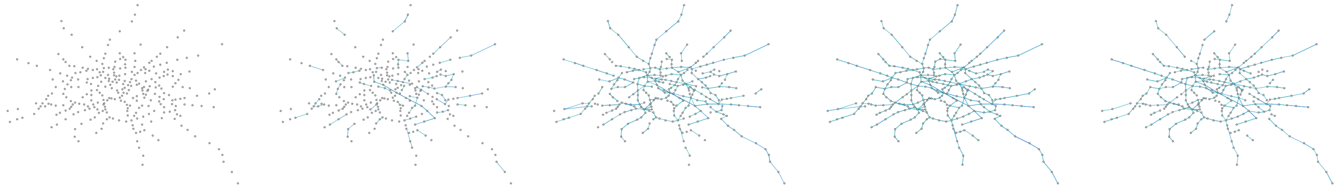

(b) Paris Metro (04:00 - 20:00 CET)

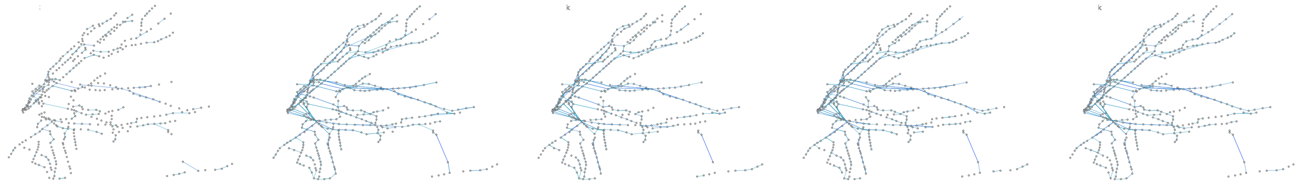

(c) New York Metro (04:00 - 20:00 EST)

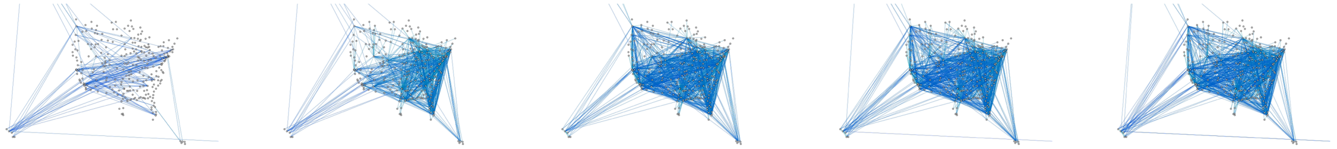

(d) US Flights (04:00 - 20:00 EST)

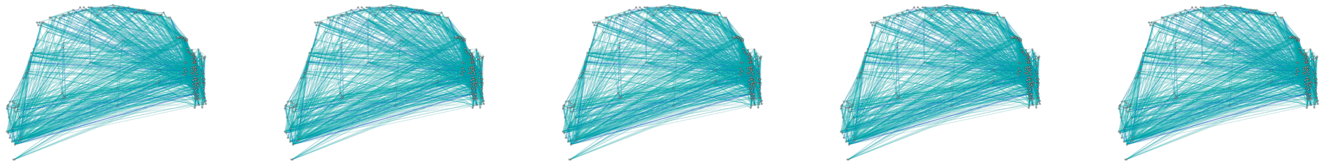

(e) C. Elegans (static)

FIG. 1. (Colour) Example snapshots in London Metro, Paris Metro, New York Metro, US Flights, and C. Elegans. StudentLife not presented due to sparsity. Edge colour represents propagation speed. Darker shades of blue indicate higher propagation speeds relative to other edges in the same network.

network geo-location. To reliably infer an individual's position from these multiple location sources we follow the same clustering approach described in Ref. [5].

c. Spatio-temporal visualisations

Fig. 1 presents example snapshots of each dataset. Each dataset's original spatial embedding is projected on to a Euclidian plane. Animations [6] of the whole duration of all five datasets can be found at `http://mattjw.net/pub/stnets`. MP4 video files are available via the Figshare Repository (doi:10.6084/m9.figshare.1452976).

d. Processed datasets

The London Metro, US Flights, Paris Metro, and New York Metro networks [7] generated for use in this paper are provided via the Dryad Repository (doi:10.5061/dryad.3p27r). Further description of the file format can be found at `http://mattjw.net/pub/stnets`. These datasets include the networks' time-varying topological and propagation information, as well as the geographic coordinates of the nodes. For privacy considerations we cannot redistribute our derivative of the StudentLife dataset, although the same spatio-temporal construction can be built from the source data. Construction of the C. Elegans network is described in the main article.

---

[1] M. Kaiser and C. C. Hilgetag, "Nonoptimal Component Placement, but Short Processing Paths, due to Long-Distance Projections in Neural Systems," PLoS Comput. Biol. **2**, E95 (2006).

[2] B. L. Chen, D. H. Hall, and D. B. Chklovskii, "Wiring Optimization Can Relate Neuronal Structure and Function," Proc. Natl. Acad. Sci. USA **103**, 4723–4728 (2006).

[3] L. R. Varshney, B. L. Chen, E. Paniagua, D. H. Hall, and D. B. Chklovskii, "Structural Properties of the Caenorhabditis Elegans Neuronal Network," PLoS Comput. Biol. **7**, E1001066 (2011).

[4] R. Wang, F. Chen, Z. Chen, T. Li, G. Harari, S. Tignor, X. Zhou, D. Ben-Zeev, and A. T. Campbell, "StudentLife: Assessing Mental Health, Academic Performance and Behavioral Trends of College Students Using Smartphones," in *Proc. 2014 Conference on Ubiquitous Computing*, UbiComp '14 (ACM, 2014) pp. 3–14.

[5] F. Tsapeli and M. Musolesi, "Investigating Causality in Human Behavior from Smartphone Sensor Data: A quasi-experimental Approach," EPJ Data Science **4** (2015).

[6] M. J. Williams and M. Musolesi, "Visualisations from: Spatio-temporal networks: reachability, centrality, and robustness," Figshare Digital Repository (2016), 10.6084/m9.figshare.1452976.

[7] M. J. Williams and M. Musolesi, "Data from: Spatio-temporal networks: reachability, centrality, and robustness," Dryad Digital Repository (2016), 10.5061/dryad.3p27r.
